# Arthroscopic C‐Shaped Release Around the Greater Trochanter for Gluteal Muscle Contracture

**DOI:** 10.1111/os.13103

**Published:** 2021-08-05

**Authors:** Xiangyu Tang, Wei Qi, Yujie Liu, Yi Xiang, Baiqing Zhang, Haipeng Li, Zhongli Li, Zhigang Wang, Dong Wang, Chunbao Li

**Affiliations:** ^1^ Department of Orthopaedics Chinese People's Liberation Army General Hospital (301 Hospital) Beijing China; ^2^ Department of Orthopaedics Chinese People's Liberation Army 985 Hospital Taiyuan China

**Keywords:** Arthroscopy, Disability, Gluteal muscle contracture

## Abstract

**Objective:**

To investigate the outcomes of C‐shaped release around the greater trochanter in gluteal muscle contracture under arthroscopy.

**Methods:**

From December 2016 to January 2018, 185 patients with gluteal muscle contracture who treated under arthroscopy were reviewed, including 69 males and 116 females. All patients had a history of repeated intramuscular injection into the buttocks. The follow signs were positive in all the patients before surgery: squatting and crouching disability, difficulty in crossing the leg, Ober's sign positive, clicking sound during rotation of the hip. The C‐shaped release around the greater trochanter under arthroscopy was performed in 96 cases (C‐shaped release group) with an average age of 24.6 ± 4.9 years old, and conventional gluteal muscle contracture release under arthroscopy was performed in 89 cases (conventional release group) with an average age of 25.1 ± 5.0 years. The released tissues in the C‐shaped release group: iliotibial band (ITB) about 5 cm distal to the proximal end of the greater trochanter, the contracture tissue near the posterior and superior of the greater trochanter, which depended on both intraoperative physical examination and arthroscopic observation. The released tissues in conventional release group: the contracture tissues in gluteal muscles according to observation under arthroscopy. The gluteal muscle contracture disability scale (GDS) and Visual analogue scale (VAS) were evaluated before surgery and at the last follow‐up.

**Results:**

The average release time after making arthroscopic operation space for each lower limb were 12.2 ± 3.2 min in the C‐shaped release group, and 21.4 ± 6.1 min in the conventional release group (*P* = 0.000). All the patients were followed for at least of 2 years after operation. There was one case of wound hematoma in the C‐shaped release group and five cases in the conventional release group(*P* = 0.079), abductor weakness (IV level)occurred in two patients in the C‐shaped release group and five cases in the conventional release group (*P* = 0.208). GDS was 49.3 ± 17.3 (22 to 70) in theC‐shaped release group and 48.1 ± 15.6 (23 to 69) in the conventional release group before surgery (*P* = 0.622), 91.7 ± 5.2 (83 to 100) in the C‐shaped release group and 90.2 ± 6.1 (83 to 98) in the conventional release group (*P* = 0.073) with difference nearly significant at last follow‐up.

**Conclusion:**

Arthroscopic C‐shaped release around the greater trochanter had less operation time, acceptable complication occurrence, and it has an optimistic outcome for gluteal muscle contracture under arthroscope.

## Graphical abstract

The target area of arthroscopic C‐shaped release was formed by the iliotibial band tissue 5 cm distal to the proximal end of the greater trochanter (A), the contracture tissue nearly posterior (B) and superior (C) of the greater trochanter.

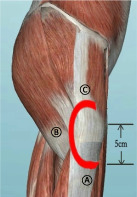

## Introduction

Gluteal muscle contracture is mostly characterized by the contracture of gluteal muscles, iliotibial band (ITB) and related fascia, the acquired gluteal contracture is generally considered to be closely related to repeated intramuscular injection of the buttocks in childhood^1^. Gluteal muscle contracture is mainly found in adolescents, and manifested as poor posture, restricted squatting and crouching, positive cross leg test, positive Ober test and positive “4” test^2^. The contractual tissue and its scope can be explored by ultrasound, magnetic resonance imaging and other examination methods^1^, ^3^. Our team first reported the arthroscopic gluteal muscle contracture release in 2009^4^, which had obvious advantages over traditional open surgery, and had been widely used as one of the main minimally invasive treatment methods^5^, ^6^.

Various types of gluteal muscle contracture were reported, cable strip contracture, fanshaped contracture, mixed contracture, and tensor fasciae latae contracture^7^. Thus, the location and degree of the contractures varied in patients, sometimes surgeons wasted time to find all the contractures in the muscles to release under arthroscopy, which could have extensive interference in normal muscle tissue, and insufficient release might happen if the operation procedure depended on limited examination and evaluation. Meanwhile, complications were sometimes reported for either arthroscopic or open surgery: muscle weakness, surgical hematoma, neurovascular injury, which related to abundant muscle tissue, regional nerve and vascular tissue in anatomy of surgical area^8^, ^9^, ^10^.

Rencently, some contracture release methods were reported to overcome the shortages of previous techniques. Rai *et al*. described a release method, including F release in ITB and gluteus maximus, and C release in deeper structures. This technique had small surgical trauma and high cosmetic satisfaction when compared to open surgery, but also had complicated procedure in respectively superficial and deeper tissue. Moreover, it had no individuation for each patient, which might lead to excessive or insufficient release^8^. Zini *et al*. used endoscopic ITB release in snapping hip, this method had simple procedure and obvious effectiveness, but not suitable for all the patients especially complicated cases, merely release the ITB might lead to insufficient release and residual symptoms in some patients^9^.

Our team designed a C‐shaped release around the great trochanter under arthroscopy. The hypothesis was according to the following three points. First, the technique was used around the great trochanter, where less muscle and neuromuscular tissue distributed, this would control the surgical complication and make the operation much safer. Second, the C‐shaped release was formed by three anatomically connected parts which were closely related to the gluteal muscle contracture in each patient, according to intraoperative physical examination and arthroscopic observation besides preoperative evaluation. This could ensure the treatment outcomes and control the rate of surgical complication. Finally, in the operation, the C‐shaped release had a constant target area, which theoretically made it easier to be operate and save time.

In this retrospective controlled study, 96 patients with gluteal muscle contracture treated with C‐shaped release around the greater trochanter, and 89 patients treated with conventional release under arthroscopy were reviewed. The purpose of study was: (i) to verify the feasibility of the release method; (ii) to explore the surgical outcomes including operating time, symptom improvement, and subjective evaluation; and (iii) to observe the complications.

## Materials and Methods

### General Information

From December 2016 to January 2018, 185 consecutive cases of gluteal muscle contracture having ITB contracture or not and treated by arthroscopic release were reviewed, including 69 males and 116 females. The inclusion criteria: (i) all patients were diagnosed with gluteal muscle contracture through medical history, symptoms and physical examination, patients with typical clinical signs including squatting and crouching disability, difficulty in crossing the legs, positive Ober's sign, snap could be detected in a passive manner; (ii) all patients underwent C‐shaped release or conventional release under arthroscopy; (iii) all patients had not responded to conservative treatment for at least 6 months, and daily lives were obviously affected; (iv) the main evaluation indicators included operation time, Gluteal muscle contracture disability scale (GDS), Visual analogue scale (VAS), and complication. This study was a retrospective case–control study.

The exclusion criteria: (i) patients with articular and extrarticular pathologies such as hip osteoarthritis, labral tears, femoroacetabular impingement syndrome, internal snapping hip, hip joint infection, and ankylosis of joint; and (ii) patients with a previous operation of the lower extremities.

From December 2016 to May 2017, 89 cases underwent conventional gluteal muscle contracture band release under arthroscopy (conventional release group). From June 2017 to January 2018, 96 cases underwent C‐shaped release around the greater trochanter under arthroscopy (C‐shaped release group). All the patients were operated on by three senior surgeons who were in the same team at our center. The function of gluteal muscle contracture was evaluated by the GDS and VAS before surgery and at the last follow‐up. The informed consent was provided by each patient in the study.

### Surgical Methods

#### The C‐Shaped Release around the Greater Trochanter

##### Anesthesia and Position

**Fig. 1:**
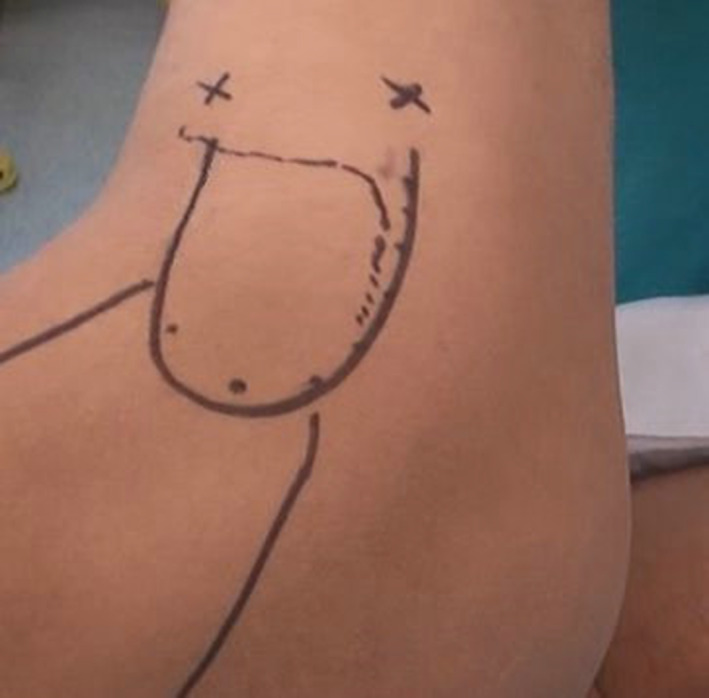
Before the surgery, delineate the scope of the greater trochanter (marked arched full line), rough outline of gluteal muscle contracture belt, two arthroscopic portals (marked +), and C‐shaped release path: ITB 5 cm nearly distal of the greater trochanter, posterior and superior of the greater trochanter.

In the C‐shaped release around the greater trochanter under arthroscopy, the lateral position was selected in all the patients, and epidural anesthesia was routinely used. Before surgery, the hip was rotated to the ideal operation posture where ITB contracture crossed over the greater trochanter with tension. In the ideal operation posture, the hip was always placed in appropriate flexion, adduction and internal rotation. The scope of the greater trochanter, rough outline of gluteal muscle contracture belt, and C‐shaped release path were delineated (Fig. 1).

##### Approach and Exposure

Two arthroscopic portals were used: anterior and posterior portals. Both of these portals are placed at 5–6 cm distal of the greater trochanter (Fig. 1). The distance of the two portals is about 5–7 cm. Then, disinfected and draped, a 5 × 5 cm operation zone was separated above the surface of ITB and gluteal muscle contracture. Saline was locally injected to form an operation space under arthroscopy, then insert the arthroscope, fat and fibrous tissues were cleared by a shaver and electrocautery device.

##### Arthroscopic Release

**Fig. 2:**
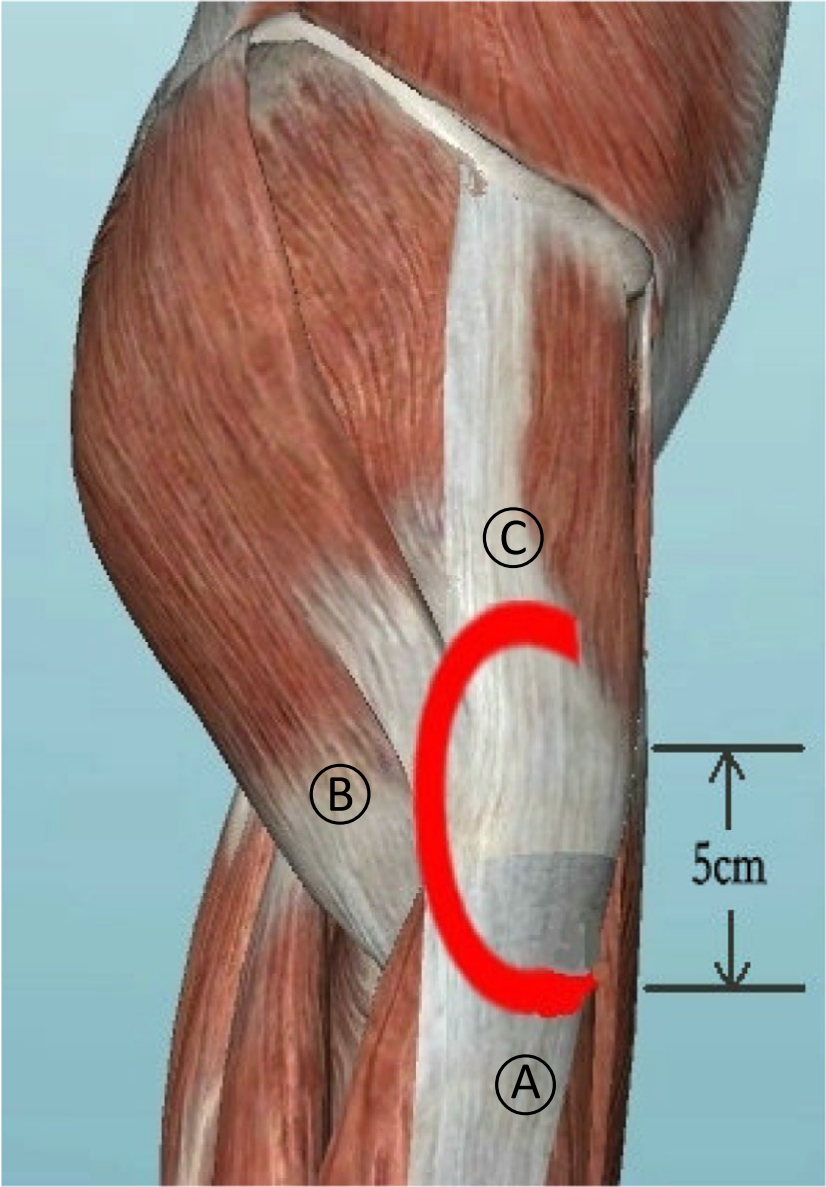
The target area of arthroscopic C‐shaped release was formed by the ITB tissue 5 cm distal to the proximal end of greater trochanter (A), the contracture tissue nearly posterior (B) and superior (C) of the greater trochanter.

The arthroscopic C‐shaped release was formed by three parts: ITB tissue about 5 cm distal to the proximal end of the greater trochanter, contracture tissue nearly posterior of the greater trochanter, and contracture tissue superior of the greater trochanter (Fig. 2). To ensure satisfactory treatment outcomes and less surgical trauma, which parts were actually released was decided according to intraoperative physical examination and arthroscopic observation.

**Fig. 3:**
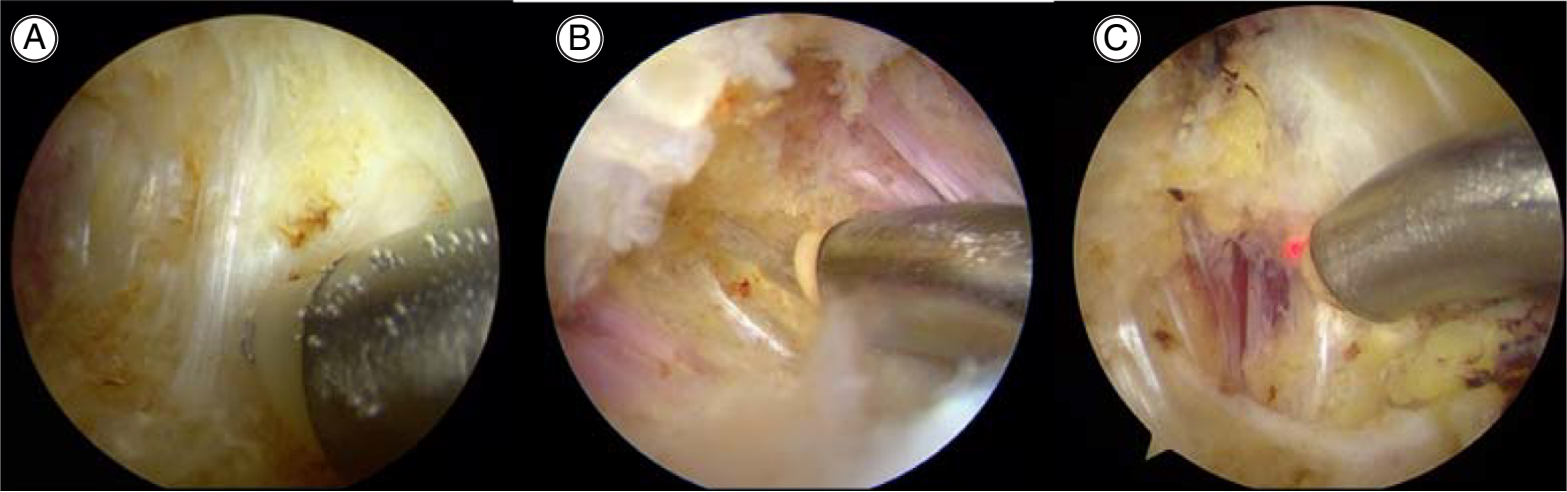
(A) In the first step of C‐shaped release, ITB tissue 5 cm nearly distal of the greater trochanter was release, which was one of key procedures in the operation. In mild patients, Ober's positive signs and main symptom disappeared after this release. (B) After the first step, if the intraoperative physical examinations were still positive, the second release step for contracture tissue nearly posterior of the greater trochanter was necessary. (C) After the second step, if there was still excessive tension of muscle contracture in intraoperative physical examinations, the contracture tissue at the superior of the greater trochanter should be released in the third step.

First, the full‐thickness ITB was transversely released at about 5 cm distal to the proximal end of greater trochanter (Fig. 3A). For patients with severe bursitis, the inflammatory tissue around the greater trochanter was debrided with a radiofrequency blade after the contracture was released. Then the intraoperative physical examination was performed, including Ober's sign, passive maximum flexion, adduction, internal rotation and abduction of the hip. If the examinations were negative, the operation procedure was finished.

If the intraoperative physical examinations were still positive, the second release step for contracture tissue near the posterior of the greater trochanter was necessary (Fig. 3B). After that, if there was still excessive tension of muscle contracture in intraoperative physical examinations, the contracture tissue at the superior of the greater trochanter was released in the third step (Fig. 3C).

After that, the intraoperative physical examination was performed again to make sure all the symptoms were negative. Next, the contralateral limb was operated in the same way.

#### The Conventional Gluteal Muscle Contracture Band Release

##### Anesthesia and Position

In conventional gluteal muscle contracture band release under arthroscopy, lateral position and epidural anesthesia was used as the C‐shaped release. The scope of the greater trochanter and rough outline of gluteal muscle contracture belt were delineated.

##### Approach and Exposure

After disinfection and draping, two or three arthroscopic portals were marked near the outline of gluteal muscle contracture belt. A 5 × 5 cm operation zone was created above the surface of ITB and gluteal muscle contracture, we introduced a motorized shaver to debride and remove fatty tissue to search for the outline of contracture bands.

##### Arthroscopic Release

Then the contracture bands were found and released until excessive tension in all the contracture bands disappeared^4^. Finally, the intraoperative physical examination was performed to ensure enough release, including Ober's sign, passive maximum flexion, adduction, internal rotation and abduction of hip. The contralateral limb was operated in the same way.

### Postoperative Treatment

After the operation, alternating lateral position was selected for compression hemostasis and wound drainage in both two groups. Postoperative analgesia and treatment for preventing myositis ossificans were given. Functional exercise was conducted 12 h after operation to prevent adhesion. Lower extremity abduction exercises were employed to strengthen gluteus muscle from 1 day to 1 year after operation.

### Outcome Measures

#### The average Release Time

In the study, the average release time calculated from after making arthroscopic operation space to the last release of the contracture.

#### Gluteal Muscle Contracture Disability Scale (GDS)

The GDS score can be used to evaluate the functional disability and recovery of the patients. All patients were evaluated before surgery and at the last follow‐up. The GDS score included gait, cross leg, squat, clicking sound, shape of buttocks, depression of local skin, walking up and down the stairs, hip tired, pain and friction of hip, pain and friction of hip, close together of knee, touch each other of ankles in supine position, restricted run, standing long jump and restricted hurdling.

#### Visual Analogue Scale (VAS)

A 10 cm horizontal line was drawn on paper, and equally divided into 10 sections. One end of the line was marked 0, indicating totally no pain; the other end was 10, indicating most severe pain; the middle sections indicated different degrees of pain. Each patient marked a certain section on the horizontal line according to the degree of pain.

#### Wound Hematoma

Wound hematoma was related to surgical injury of muscle and fat tissue, which would delay the recovery time. In the study, we did not use drainage for all patient, this allowed patients early functional exercise.

#### Abductor Weakness

Excessive release of the muscle would result in abductor weakness. The normal muscle tissue should be wounded as little as possible during the operation. In the C‐shaped release, the area around the greater trochanter had less muscle tissue to be released than the release area in the conventional technique, which meant less effect on muscle strength.

### Statistical Method

We used SPASS 22.0 software (IBM, Armonk, NY, USA) for statistical analysis. Quantitative data included age, the average release time, GDS and VAS, which were tested by the K‐S normal distribution test and variance homogeneity test showed normal distribution and neat variance. A *t*‐test was used to compare the age, the average release time, the GDS and VAS before and after surgery of the two groups. Count data included gender, the number of wound hematoma and abductor weakness in the groups. Comparisons between counts were compared by a chi‐square test. Statistical differences were considered significant for *P* values < 0.05.

## Results

### General Results

All the patients had a history of repeated intramuscular injection of drugs into bilateral buttocks. There were 35 males and 61 females in the C‐shaped release group, with an average age of 24.6 ± 4.9 years. There were 34 males and 55 females in conventional release group, with an average age of 25.1 ± 5.0 years. There was no statistically significant difference in preoperative general conditions between the two groups (table 1).

**TABLE 1:** Patients underwent arthroscopic C‐shaped release around greater trochanter (C‐shaped release group) *vs* conventional gluteal muscle contracture band release (conventional release group)

Parameters	C‐shaped release group	conventional release group	*t*/*χ* ^*2*^	*P*
Gender (male/female)	35/61	34/55	*χ* ^*2*^ = 0.237	0.627
Age (years old)	24.6 ± 4.9	25.1 ± 5.0	*t* = 0.687	0.493
Operation time (mins)	12.2 ± 3.2	21.4 ± 6.1	*t* = 12.978	0.000 *
Wound hematoma (cases)	1/96	5/89	*χ* ^*2*^ = 3.082	0.079
Abductor weakness (cases)	2/96	5/89	*χ* ^*2*^ = 1.585	0.208
Preoperative GDS	49.3 ± 17.3	48.1 ± 15.6	*t* = 0.494	0.622
GDS at last follow‐up	91.7 ± 5.2	90.2 ± 6.1	*t* = 1.804	0.073
Preoperative VAS	3.5 ± 1.6	3.6 ± 1.8	*t* = 0.400	0.690
VAS at last follow‐up	0.052 ± 0.22	0.067 ± 0.25	*t* = 0.434	0.665

GDS, gluteal muscle contracture disability scale; VAS, Visual analogue scale.

^*^ Meant *P* < 0.05.

### Feasibility and Surgical Outcomes

The average release time after making arthroscopic operation space for each lower limb was 12.2 ± 3.2 min (range, 6 min to 21 min) in the C‐shaped release group and 21.4 ± 6.1 min (range, 11 min to 36 min) in the conventional release group (*P* < 0.05). No major neurovascular injury occurred during the operation, and the sensory and motor functions of both lower limbs were normal after the operation. One case remained with squatting difficulty in the conventional release group, the symptom disappeared after functional exercise. In all other patients, there was no difficulty in squatting or raising legs, and Ober's disease and hip bounce were negative (Cases 1–2).

### Gluteal Muscle Contracture Disability Scale (GDS)

The follow‐up time was more than 2 years (range, 2 to 3 years), and GDS before surgery was 49.3 ± 17.3 (range, 23 to 70) in the C‐shaped release group and 48.1 ± 15.6 (range, 22 to 69) in the conventional release group, with no statistically significant difference (*P* > 0.05). At the last follow‐up, the GDS were 91.7 ± 5.2 (range, 84 to 100) in the C‐shaped release group and 90.2 ± 6.1 (range, 83 to 98) in the conventional release group, the difference was nearly significant (*P* = 0.073) (table 1).

### Visual Analogue Scale (VAS)

VAS before surgery was 3.5 ± 1.6 (range, 0 to 6) in the C‐shaped release group and 3.6 ± 1.8 (range, 0 to 6) in the conventional release group, with no statistically significant difference (*P* > 0.05). At the last follow‐up, the VAS was 0.052 ± 0.22 (range, 0 to 1) in the C‐shaped release group and 0.067 ± 0.25 (range, 0 to 1) in the conventional release group (Table 1).

### Complications

There was no revision surgery. In the C‐shaped release group, one case had temporary wound hematoma after operation, and five cases in the conventional release group, the statistical difference was nearly significant (*P* = 0.079). Two cases had abductor weakness of hip joint (muscle strength grade IV) in the C‐shaped release group, and five cases in the conventional release group (*P* = 0.208), due to excessive release, who had near normal muscle strength before surgery. At the last follow‐up, muscle strength of the six cases returned to normal. All patients had good wound healing (Table 1).

## Discussion

In this study, we introduced an arthroscopic C‐shaped release around greater trochanter in gluteal muscle contracture, which had optimistic outcomes on operating time, symptom improvement, complications, and GDS score according to this comparative study.

### Basic Background

Gluteal muscle contracture was first reported by Valderrama in 1969^11^. It was characterized by contracture of gluteal muscles, tensor fascia lata, ITB, *et al*.^12^, ^13^, ^14^. Patients were found all over the world, but many more were reported in China with a childhood incidence rate of 1%–2.5%^1^. For the refractory patients who did not response well to the nonsurgical treatments, surgical intervention was necessary. Arthroscopy release in gluteal muscle contracture was first reported by our team, and had obtained a good surgical effect^4^. Then Rai *et al*. and Zhang *et al*. respectively introduced the advantage of arthroscopic surgery by comparing the arthroscopic surgery and open surgery^8^, ^15^.

### Feasibility of the Release Method

Some surgeons reported several methods for release contracture band. Rai *et al*. reported a method with F release in ITB and gluteus maximus contractures, and C release in deeper structures, which had a complicated procedure and no individuation for each patient^8^. Zini *et al*. used endoscopic ITB release in snapping hip, however this invariant release way may lead to insufficient release in complex cases^9^. Other surgeons performed small incision surgery around the greater trochanter following a special designed pathway to release contracture bands^16^. In this study, we used C‐shaped release around the great trochanter under arthroscopy in order to obtain better outcomes. The C‐shaped area around the greater trochanter, was surrounded by less muscle tissue to be released under arthroscopy (Fig. 2), which theoretically meant less effect on muscle strength. Meanwhile, this area did not have known nerve and vascular tissue, which reduced the rate of neurovascular injury.

Our team described various types of contracture under arthroscopy in 2013^7^, which provided more reference information for arthroscopic release in gluteal muscle contracture. Different from merely referring to preoperative evaluation in some of the previous studies, this study carefully evaluated the intraoperative physical examination and arthroscopic observation beside preoperative evaluation, to avoid insufficient release and poor clinical outcomes. In the ‘C' shape release around the greater trochanter, we routinely transversely released the ITB at about 5 cm distal to the proximal end of the greater trochanter at first, which was one of key procedures in the operation. In mild patients, Ober's positive signs and main symptom disappeared after this release, which was mentioned in Zini *et al*.'s report^9^. If clicking sound and excessive muscle tension of the hip existed after the above release, the second and third steps should be performed closely according to the arthroscopic observation, longitudinal release of the contracture tissue near the posterior the greater trochanter or additional release of the tissue near the superior of the greater trochanter. This C‐shape release was limited to the area around the great trochanter which theoretically was easier to operate.

### Surgical Outcomes

At the same time, the arthroscopic contracture release time after making arthroscopic operation space for each lower limb in C‐shaped release was 12.2 min ± 3.2 min, and 21.4 min ± 6.1 min (*p* < 0.05) in the conventional release group, which showed that C‐shaped release around the greater trochanter was more timesaving. In this study, the difference of GDS between patients with C‐shaped release around the greater trochanter and patients with conventional release under arthroscopy was not significant, but the difference was nearly significant in GDS at last follow‐up. Thus arthroscopic C‐shaped release around the greater trochanter in gluteal muscle contracture count achieved optimistic outcomes with few complications.

### Complications

In this study, one patient had transient wound hematoma in the C‐shape release group and five patients in the conventional release group under arthroscopy, the statistical difference was nearly significant (*p* = 0.079). Simultaneously, two patients had abductor weakness in the C‐shape release group and five patients in the conventional release group under arthroscopy (*p* = 0.208).

### Shortcomings of the Study

Meanwhile, there were several limitations in the study. First of all, the impairment of muscle tissue during C‐shaped release and conventional gluteal muscle contracture release under arthroscopy was not evaluated. From the anatomy point of view, C‐shaped release had less interference on soft tissue, especially muscle tissue. Second, the ITB was released for all the patients in the study, which might have impact on lower limb alignment. Although we did not find any obvious symptoms on hip, knee or ankles in the patients as Zini *et al*.'s study^9^, further study on lower limb alignment and longer follow‐up time should be made. Third, this was a retrospective study, further prospective studies should be conducted to further verify the validity of the technique in the next long‐term follow‐up study.

In conclusion, according to this comparative study, arthroscopic C‐shaped release around the greater trochanter for gluteal muscle contracture was a safe, effective technique. It could shorten the operation time and reduce the difficulty of arthroscopic release when compared to the conventional technique under arthroscopy.

Case 1 Male, 22 years old, gluteal muscle contracture, the cross leg test was positive before surgery (A), and negative after arthroscopic C‐shaped release around greater trochanter (B).
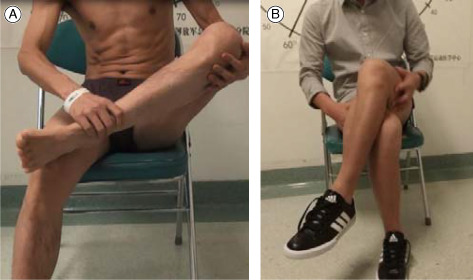

Case 2 Male, 19 years old, gluteal muscle contracture, the squatting and crouching was difficulty before operation (A), and easy after arthroscopic C‐shaped release around greater trochanter (B).
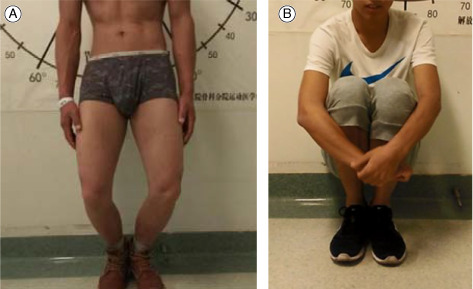

## Gluteal muscle contracture disability scale (GDS)

**Table:**

**Items**	**Score**
1. Trendelenburg gait	
Very seriously walk restricted	1
Obviously sports restricted	4
Mild abnormal gait	7
Normal	10
2. Cross leg	
Absolutely cannot	1
Ankle can only reach the opposite knee joint	4
Calf can only reach the opposite knee joint	7
Normal	10
3. Squat with knees closing together	
Absolutely cannot	1
Restricted, can complete on tiptoe	4
Slightly restricted, can only complete with both feet together and whole sole of foot on floor	7
Normal	10
4. Clicking sound of hip	
Happen when walk or squat	1
Happen when squat, but not walk	4
Happen only when the hip in internal rotation, adduction and flexion position, but not walk or squat	7
Not happen	10
5. Shape of buttock	
Pointed shape	1
Flat shape	4
Mild atrophy of buttock muscle	6
Normal	8
6. Depression of local skin	
Serious depression in any hip position	1
Obvious depression in hip adduction and flexion	4
Mild depression when squat	6
Normal	8
7. Walking up and down the stairs	
Restricted	0
Normal	2
8. Hip tired	
In daily life	1
In moderate sports	2
Only in strenuous sports or long time squat	3
Normal	4
9. Pain and friction of hip	
In daily life	1
In moderate sports	2
Only in strenuous sports or long time squat	3
Normal	4
10. Sitting posture	
Cannot stay upright posture	0
Can stay upright posture only when knees apart	2
Can stay upright posture with knees closing together	6
11. Knees can close together with lower limbs extension in lateral position	
Cannot	0
Can	10
12. Ankles can touch each other with lower limbs extension and cross in supine position	
Cannot	0
Can	10
13. Run was restricted	
Yes	0
No	2
14. Standing long jump	
Difficulty	0
Easy	2
15. Hurdling was restricted	
Very seriously	1
Obviously	2
Mildly	3
Normal	4
